# Impressive Results after “Metabolism-Guided” Lattice Irradiation in Patients Submitted to Palliative Radiation Therapy: Preliminary Results of LATTICE_01 Multicenter Study

**DOI:** 10.3390/cancers14163909

**Published:** 2022-08-12

**Authors:** Gianluca Ferini, Silvana Parisi, Sara Lillo, Anna Viola, Fabio Minutoli, Paola Critelli, Vito Valenti, Salvatore Ivan Illari, Anna Brogna, Giuseppe Emmanuele Umana, Giacomo Ferrantelli, Gabriele Lo Giudice, Chiara Carrubba, Valentina Zagardo, Anna Santacaterina, Salvatore Leotta, Alberto Cacciola, Antonio Pontoriero, Stefano Pergolizzi

**Affiliations:** 1 REM Radioterapia srl, 95029 Viagrande, Italy; 2 Department of Biomedical, Dental Science and Morphological and Functional Images, University of Messina, 98122 Messina, Italy; 3 Fondazione IOM, 95029 Viagrande, Italy; 4 Medical Physics Unit, A.O.U. “G. Martino”, 98122 Messina, Italy; 5 Department of Neurosurgery, Trauma Center, Gamma Knife Center, Cannizzaro Hospital, 95126 Catania, Italy; 6 Radiation Oncology Unit, Papardo Teaching Hospital, 98158 Messina, Italy

**Keywords:** palliation, lattice radiation therapy, bulky tumors

## Abstract

**Simple Summary:**

The lattice technique is a particular form of spatially fractionated radiation therapy, which was demonstrated to be safe and effective for treating advanced cancers. Bulky tumor disease is difficult to treat. In this clinical scenario, radiotherapy has a role in the palliation of symptoms. The lattice technique allows high doses to be delivered within tumor masses. Combining lattice delivery with IMRT/VMAT to bulky tumors offers optimal symptom control and could emerge as the best therapeutic option in this setting of patients.

**Abstract:**

Purpose: To evaluate feasibility, toxicities, and clinical response in Stage IV patients treated with palliative “metabolism-guided” lattice technique. Patients and Methods: From June 2020 to December 2021, 30 consecutive clinical stage IV patients with 31 bulky lesions were included in this study. All patients received palliative irradiation consisting of a spatially fractionated high radiation dose delivered in spherical deposits (vertices, Vs) within the bulky disease. The Vs were placed at the edges of tumor areas with different metabolisms at the PET exam following a non-geometric arrangement. Precisely, the Vs overlapped the interfaces between the tumor areas of higher ^18^F-FDG uptake (>75% SUV max) and areas with lower ^18^F-FDG uptake. A median dose of 15 Gy/1 fraction (range 10–27 Gy in 1/3 fractions) was delivered to the Vs. Within 7 days after the Vs boost, all the gross tumor volume (GTV) was homogeneously treated with hypo-fractionated radiation therapy (RT). Results: The rate of symptomatic response was 100%, and it was observed immediately after lattice RT delivery in 3/30 patients, while 27/30 patients had a symptomatic response within 8 days from the end of GTV irradiation. Radiation-related acute grade ≥1 toxicities were observed in 6/30 (20%) patients. The rate of overall clinical response was 89%, including 23% of complete remission. The 1-year overall survival rate was 86.4%. Conclusions: “Metabolism-guided” lattice radiotherapy is feasible and well-tolerated, being able to yield very impressive results both in terms of symptom relief and overall clinical response rate in stage IV bulky disease patients. These preliminary results seem to indicate that this kind of therapy could emerge as the best therapeutic option for this patient setting.

## 1. Introduction

Palliative radiation therapy in advanced/metastatic cancer patients is devoted to control symptoms, to relieve pain or bleeding, to maintain an adequate quality of life, and in some instances, to ameliorate the overall survival by facilitating systemic treatments in controlling a lesser disease burden. In palliative settings, irradiation has to be delivered in a judicious manner. Indeed, the radiation oncologist has to balance the pros and cons of palliative intent radiation therapy to maintain a favorable therapeutic ratio: treatments should not cause either acute or late sequelae in already suffering patients. In the management of advanced/metastatic incurable cancer with radiotherapy, historical results showed that good symptomatic palliation is obtained using hypo-fractionated irradiation delivering 20 Gy in 4–5 fractions or 30 Gy in 10 fractions [1]. With this kind of treatment, it is possible to obtain adequate palliation without the alteration of patients’ quality of life due to a low toxicity profile and a rapid onset of symptom control. In addition, the low number of hospital accesses does not occupy time in patients with short life expectancy. Bulky disease in solid cancers presents many challenges when the purpose is to deliver adequate radiation doses using large irradiation fields. Spatially fractionated radiation therapy (SFRT) is an irradiation technique that allows the delivery of high doses to small volumes [2], and some papers seem to demonstrate its feasibility in palliative settings [3,4,5,6]. A particular form of SFRT is the lattice technique, which has the particularity to deliver a high radiation dose to so-called “vertices” while maintaining a low dose to the gross tumor volume (GTV) periphery; this permits the preservation of peritumoral lymphocytes that could help to activate an immunological reaction against cancer cells, and so, we might use the potential of radiotherapy to mobilize a systemic immune-mediated tumor response [7]. Moreover, such a peculiar dose delivery method could counteract the typical non-homogeneous tumor growth by selecting different metabolic areas (which could include both hypoxic regions and different tumor microenvironments) to be boosted to overcome their relative radio-resistance [8,9]. A therapeutic option is to differentiate the radiation dose delivery according to the local distribution of oxygenated areas: this is so-called “oxygen-guided radiotherapy”, a new investigational approach not yet reported in large clinical trials [10]. The ^18^F-FDG uptake could be higher in hypoxic cancer cells than normoxic cancer cells; this finding has been reported both in in vitro [11] and in in vivo studies [12]. In addition, non-classical radiation doses provoke radiation-induced tumor cells’ damage with the production of tumor antigens and molecular products with a secondary activation of antigen-presenting cells and T-lymphocytes. The immune activation is defined as “in situ” radio-vaccination [13]. Finally, high radiation doses stimulate some mechanisms in the tumor microenvironment [14,15], which permit bystander, abscopal, and immunological effects to be activated. According to this background, we decided to treat small subvolumes (vertices) astride the different metabolic areas of bulky tumor masses. Here, we report the clinical results derived by the implementation of a “metabolism-guided” vertex placement in the lattice technique to treat patients with metastatic bulky disease.

## 2. Patients and Methods 

### 2.1. Eligibility Criteria and Pretreatment Evaluation

Patients with >5 cm masses developing from solid cancers (bulky disease) in clinical stage IV and age >18 years were considered to be eligible. Other requirements for eligibility were an ECOG Performance Status ≤2, a life expectancy >2 months, and the absence of a clinical diagnosis of superior vena cava syndrome, extradural spinal compression, and severe bleeding. Patients with a history of previous irradiation to the bulky disease site were excluded. All patients received an “internal multicenter study group” recommendation for radiotherapy with or without chemotherapy, or immunotherapy, hormone therapy, or targeted therapy. Patients who received or planned to receive chemotherapy or immuno-/targeted therapy before or after lattice SFRT were eligible on the condition that an arbitrarily defined 5-day break for drug washout was observed.

The pretreatment evaluation included: physical examination and complete blood count; head, neck, thoracic, and superior/inferior abdomen computed tomography (CT); [^18^F]-fluorodeoxyglucose positron emission tomography/computed tomography (^18^F-FDG-PET/CT); and an MRI study was carried out when needed. Other investigations were conducted in the presence of clinically suspect signs. We considered every lesion unsuitable for surgical resection or ablative stereotactic irradiation with ^18^F-FDG uptake on the whole body PET/CT scan as bulky disease.

The treatment described in this article was delivered to patients on the basis of their clinical circumstances, without the specific testing of a research hypothesis. All the patients received a detailed explanation of the treatment risks from the treating radiation oncologist and provided written informed consent to treatment. The present observational study, performed prospectively and named LATTICE_01, was approved by Messina Ethics Committee with protocol number 1611-38-21. 

### 2.2. Treatment

#### 2.2.1. Vertex Positioning and Treatment Planning for Bulky Disease

**Figure 1:**
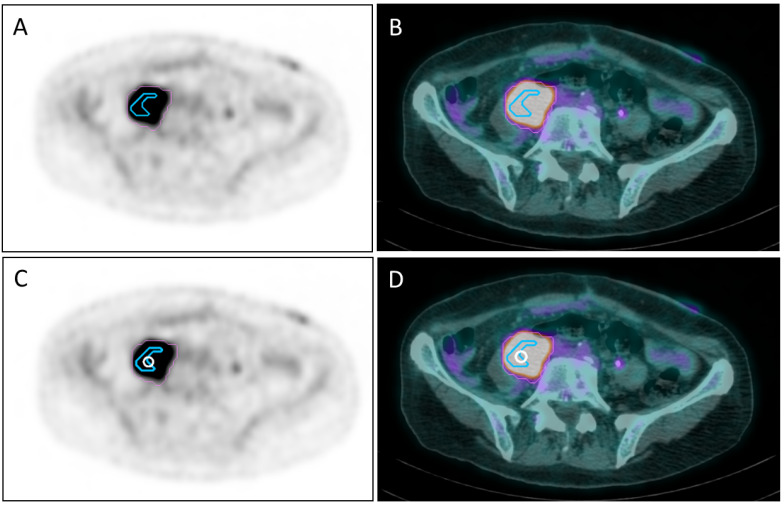
PET (**A**) and PET/CT (**B**) images showing the segmentation of the metabolic activity of a retroperitoneal mass with SUVmax = 13.31: the pink line delimits the “avid” PET area (APA) with SUV >2.5, whereas the light blue line delimits a “super-avid” PET area (SAPA) with SUV >75% SUVmax of APA. The white circle on the same PET (**C**) and PET/CT (**D**) slices of (**A**,**B**) represents a 1 cm-diameter sphere called “Vertex” placed between SAPA and the remaining part of APA.

Within the tumor mass we defined: 1. a “Photopenic PET Area” (PPA) showing lower activity than the blood pool and corresponding to a low-density (sub-solid) area on CT images, suggesting a necrotic core; 2. an “Avid” PET Area (APA) with SUV > 2.5; 3. a “Super-Avid” PET Area (SAPA) as the part of APA showing SUV >75% SUVmax. We attributed such differences to a heterogeneous oxygen landscape within the tumor, and we positioned a 1 cm-diameter sphere called “Vertex” between the SAPA and the remaining part of the APA (Figure 1). When the SAPA was almost coincident with the APA, and a PPA was present, the vertex was positioned between the PPA and APA. This choice was arbitrary because there is not any theory supported by evidence on this issue; however, regions with different SUVs within a bulky mass could have different rates of cell growth, oxygenation, and tumor microenvironment. The number of spheres (median 4, range 1–6) was chosen arbitrarily by each radiation oncologist participating in the study, according to the neighboring organs at risk (OARs) and mass volume: caution was exercised to avoid placing high-dose vertices in or close to neural structures, large vessels, and bones. In addition, vertices had to have at least a 2.0 cm (center to center) distance from each other. The vertices had no geometrically defined arrangement in an analogous way, as previously carried out by Tubin et al. [16]. On day 1, all patients had stereotactic/Intensity Modulated Radiation Therapy (IMRT)/Volumetric Modulated Arc Therapy (VMAT) delivered to the vertices using the following Linear Accelerators (LinAc): A. True-beam (Varian, Palo Alto, CA, USA); B. Agility (Elekta, Stockholm, Sweden); C. Synergy (Elekta, Stockholm, Sweden); D. robotic-arm linear accelerator (Cyberknife, Sunnyvale, CA, USA). All not-dedicated LinAcs were not specifically designed for the lattice technique; they were equipped with cone-beam-CT (with the exception of D) and A, B, and D also had a *6D*-Robotic-Couch (Brainlab^®^, Munich, Germany).

A CT simulation (1.25 mm thickness slices) was used for treatment planning and was co-registered with the ^18^F-FDG-PET-CT. In most cases, an MRI simulation scan was performed and was merged with the planning CT scan with IV contrast for accurate tumor delineation. The Bulky-Gross Tumor Volume (B-GTV) was delineated. Additionally, a Clinical Target Volume (CTV) was determined around the GTV on a case-by-case basis to duly take into account the possible subclinical tumor spread. The PTV definition varied according to the LinAc equipment (including tumor motion tracking) ranging from 2 to 12 mm of isotropic margin expansion, which could be manually trimmed to spare any neighboring critical OARs. Set-up verification was conducted daily with orthogonal anterior–posterior/lateral low-energy X-rays/MV pair and cone beam CT. The prescription of the radiation dose was performed according to the International Commission on Radiation Units & Measurements (ICRU) recommendations and with due regard for the dose-volume constraints suggested by the Quantitative Analyzes of Normal Tissues Effects in the Clinic (for dose fraction <5 Gy) and Hanna et al. [17] for dose fraction ≥5 Gy. The planned dose to be delivered to the vertices was at least 10 Gy/1 fraction, and the optimized plan had to result in ≥98% dose coverage of the vertices volume. 

Patients were followed-up with contrast-enhanced CT 30 and 60 days after the completion of radiotherapy; afterwards, if no suspected progressive disease was detected, CT and/or CT/PET scans were performed every 3 months for the first year and every six months after.

#### 2.2.2. Treatment Scheme

On day 1, a high radiation dose was delivered to the vertices. Within 7 days, a homogeneous PTV irradiation started with a total dose prescription based on the radiation oncologist’s preferences.

### 2.3. Endpoints

We accrued a consecutive series of patients to obtain a consistent population with precisely defined characteristics and homogeneous treatment. The primary endpoints were symptomatic response, radiation-therapy-related early and late toxicities evaluation, and local control rate. The overall survival (OS), the time from primary treatment to death, was also evaluated as a secondary endpoint. 

#### Evaluation of Toxicities and Response Assessment 

Toxicity was evaluated using the Radiation Therapy Oncology Group (RTOG) criteria and the Common Terminology Criteria for Adverse Events (CTCAE) version 4.1. Acute toxicity was defined as toxicity occurring during or at the end of radiation therapy or within 60 days from the end of irradiation. Symptomatic response was defined as the subjective amelioration of symptoms (pain, moderate bleeding, and annoying swelling). The pain score was evaluated using the visual analog scale (VAS). This consists of a 10 cm straight line with the endpoints defining extreme limits such as “no pain” and “worst pain”; the following descriptive terms, mild, moderate, and severe, were added to the VAS. The symptomatic response included three settings: complete response (CR), defined as the complete resolution of symptoms; partial response (PR), defined as some symptomatic improvement; and no response (NR) for no symptomatic improvement. Clinical response was defined according to the clinician’s point of view: palpable mass/node shrinking and initial or definitive ulcer healing. Clinical evaluation was performed at the beginning and end of treatment, and subsequently, at 8, 30, and 60 days after the completion of radiation therapy. Chronic toxicity was defined as toxicity occurring after 90 days from the end of irradiation, or when acute reactions persisted over 90 days. The Response Evaluation Criteria in Solid Tumors, version 1.1, and/or PET/CT Response Criteria in Solid Tumors were used to evaluate treatment efficacy. The overall survival (OS) was defined as the time from the start of RT until reported death due to any cause or censoring by the date of last follow-up if the patient was alive. Local control (LC) was defined from the start of RT until local progression or last imaging available. OS distribution was estimated according to the Kaplan–Meier method. 

## 3. Results

**Figure 2:**
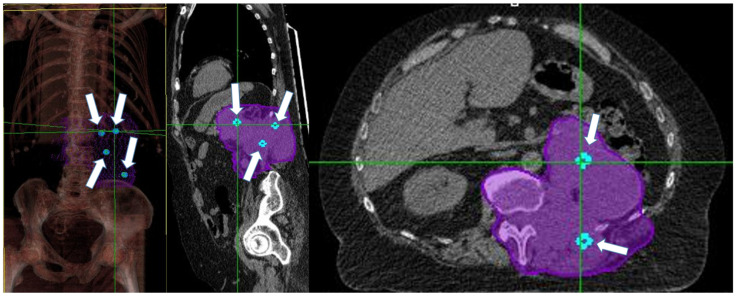
White arrows show Vs (light-blue spheres) position in a patient with retroperitoneal bulky disease. Purple encloses the entire gross tumor volume and the adjacent vertebra (CTV).

Between June 2020 and December 2021, thirty patients were enrolled. As a patient was irradiated in 2 disease sites, the total number of treated tumor sites was 31. There were 10 female and 20 male patients with a median age of 74.5 years (range 42–91) and a median ECOG status of 2 (range 0–2). Primary tumor sites in 30 patients were: six lung (20%), five soft tissue (16.6%), three bladder (10%), three kidney (10%), three skin (10%), two uterus (6.7%), two breast (6.7%), two ureter (6.7%), two rectum (6.7%), one penis (3.3%), and one unknown primary site (3.3%). In 13/30 patients, the bulky disease also involved the bone. Table 1 shows patients and tumors characteristics. All patients had a mass with at least an axial dimension >5cm; median GTV was 146, 48cc (range 50.9–2039.7cc). A median of 4 Vs (range 1–6) was placed within the GTV. Figure 2 shows an example of Vs placement. A median dose of 15 Gy/1 fraction (range 10 Gy/1 fr–27 Gy/3 fractions) was delivered to the Vs. The median dose delivered to the PTV was 20 Gy/4 fractions (range 18 Gy/3 fr–40.05 Gy/15 fr). The treatment of PTV started within 7 days after Vs irradiation. 

Table 2 shows treatment characteristics. Median follow-up time was 10.75 months (range: 6.8–20.5 months), with 23 patients being still alive and 3 patients lost to follow-up.

All patients showed symptomatic response at the treatment site (31 sites in 30 patients) during irradiation. Acute toxicities were observed in nine patients: one had G2 mucositis, two had G1 dysphagia, five had G1 skin toxicity, and one had G1 gastrointestinal toxicity (diarrhea). During irradiation, 30/30 patients showed some treatment benefit; in particular, 3 patients showed pain disappearance 2 days after Vs irradiation. On the eighth day from the end of radiation therapy, a median VAS score of 1.5 was observed (range 0–4) with descriptive terms “mild” and “moderate” in 11 and 20 treated sites. No flare pain has been reported. From a clinical point of view, response was complete in 11 patients and partial in 4 patients with palpable mass/nodes; definitive ulcer healing has been reported in ¾ patients with this clinical scenario. One patient showed telangiectasia as chronic cutaneous toxicity. In the imaging follow-up studies, one in-field disease progression was reported. A complete regression was observed in 5/30 patients, while 24 patients showed a partial regression. Table 3 summarizes the responses to treatment and radiation toxicities. Symptomatic relapse was reported in two patients who were submitted to re-irradiation. Six- and 12-month overall survival was 86.4%. 

## 4. Discussion

In our study, we used a particular form of the lattice approach, which presents differences with respect to the “classic” SFRT techniques [18]. In fact, we did not follow a “geometric” pattern for Vs positioning within the GTV: Vs were placed looking at the differences in ^18^F-FDG-uptake within the bulky disease; this choice was justified by the fact that the ^18^F-FDG uptake could be higher in hypoxic than normoxic cancer cells; this finding has been reported both in in vitro [11] and in in vivo studies [12]; on the other hand, the low-^18^F-FDG-uptake portion of a tumor may not indicate the lack of viable cancer cells [12]. We observed low toxicity profiles and impressive clinical results. Actually, we reported a symptomatic response in 100% of patients, maintaining long-lasting symptom control in 28/30 patients. These results confirm both the low toxicity profile and clinical response observed in other published case series and case reports [19,20,21,22]. It is noteworthy to underline that in our patients, we observed a rapid symptomatic response after the first day of irradiation (radiotherapy to the vertices) in 3/30 patients, while a symptomatic benefit with a reduction in VAS score was reported within 8 days from the end of irradiation in all treated patients. These observations seem to confirm both the efficacy of our approach for treating bulky tumors and the results reported by Duriseti et al. [5], who reported a symptomatic response within 14 days from the start of radiotherapy in 20 patients with 22 lesions. Surprisingly, in our patients with bone involvement, we did not observe the occurrence of flare pain, which has been reported to occur in 2–44% of patients with bone metastases after irradiation [23]. We are unable to explain this issue. A complete clinical response in patients with palpable mass/nodes was obtained in 11 patients, confirming the rapid onset of response using lattice RT delivery; in addition, definitive ulcer healing was obtained in 75% of patients presenting this clinical scenario. Similarly, imaging studies confirmed a complete or partial regression of bulky disease in 30/31 sites in 29/30 patients. These data also confirm what has been reported in other published series [4,20,21,24,25]. It is worth noting the high rate of the long-term local control of disease, which was reported in almost all cases (29/30 patients) with only two symptomatic relapses that required a second course of irradiation. These observations have not previously been reported. The choice to place the Vs on the border between different metabolic areas (plausibly reflecting different oxygenation) should be better defined using different tools (i.e., [^18^F]FMISO PET/CT) [10] than ^18^F-FDG-PET, and this is a limitation of our study. 

We are fully aware that this study has several limitations, including the miscellaneous collection of treated tumors, the definition of different metabolic areas, and the arbitrary positioning of vertices within the bulky disease. In spite of the latter, we observed similar responses, which seemed to be independent from the personal considerations of each treating radiation oncologist involved here in choosing the most convenient Vs placement. This is the strength of the study. Another limitation of the study may be related to the assessment of clinical response from the point of view of the observing physician; this may represent a bias for the study. However, this kind of therapy for bulky tumors may move radiation therapy from a palliative intent focusing on short-term improvements in patients’ quality of life towards an effort to achieve long-term tumor control. 

These findings should stimulate a collaboration between clinicians and basic scientists, as insight into a therapeutic application should be converted into studies in molecular biology, cell biology, and biochemistry: a reverse process, “from bedside to bench”. In fact, according to Bing Gan (a plastic surgeon): “A clinician may not be the best person to answer a question, but may be the best person to ask a question.” [26].

## 5. Conclusions

“Metabolism-guided” lattice radiotherapy is feasible and well-tolerated, being able to yield very impressive results both in terms of symptom relief and overall clinical response rate in stage IV bulky disease patients. These preliminary results, obtained by combining vertex irradiation with a dose capable of producing bystander, abscopal, and immunological effects with the treatment of GTV using classical palliative doses, seem to indicate that this kind of therapy could emerge as the best therapeutic option for this patient setting.

## Figures and Tables

**Table 1:** Patient and tumor characteristics.

Age (years)	
Median	74.5
Range	42–91
**ECOG Status**	
Median	2
Range	0–2
**Sex**	
Female	10
Male	20
**Tumor-Related Symptoms**	
Pain	24
Neuropathic pain	3
Palpable mass	5
No pain	3
**VAS Score**	
Median	5
Range	0–10
**Histology**	
Adenocarcinoma	8
Squamous cell carcinoma	7
Urothelial carcinoma	5
Soft tissue sarcoma	5
Ductal carcinoma	2
Malignant melanoma	3
**Bulky Disease Location in 31 Sites/30 Patients**	
*Head and neck*	4
*Trunk*	
- Intrathoracic	5
- Abdomen–pelvis	15
- Breast	2
- Soft tissue	4
*Lower extremities*	1
**Tumor size in 31 Sites/30 Patients**	
5–10 cm	25
>10 cm	6
**Gross Tumor Volume, cc**	
Median	146,8
Range	50.9–2039.7

**Table 2:** Treatment characteristics.

Systemic Therapy Immediately Preceding Irradiation	
Chemotherapy	17
Immunotherapy	3
Chemo-Immunotherapy	6
None	4
**Dose Fraction to Vertices in 31 Sites/30 Patients**	
Median	15 Gy/1 fx
10 Gy/1 fx	12
15 Gy/1 fx	13
18 Gy/1 fx	1
21 Gy/3 fx	1
24 Gy/3 Fx	1
27 Gy/3 Fx	3
**Dose-Fraction Schemes to GTV in 31 Sites/30 Patients**	
Median	20 Gy/4 fx
18 Gy/3 fx	1
20 Gy/4 fx	17
22.4 Gy/4 fx	1
30 Gy/3 fx	10
30 Gy/5 fx	1
40.5 Gy/15 fx	1

**Table 3:** Response to treatment and radiation toxicities.

Symptomatic Benefit after Irradiation	
Yes	30
No	0
**VAS Score**	
Median	1.5
Range	0–4
**Acute Toxicity**	
None	21
G2 mucositis	1
G1 dysphagia	2
G1 skin	5
G1 diarrhea	1
**Late Toxicity**	
None	29
Skin	1

## Data Availability

The data presented in this study are available on request from the corresponding author. The data are not publicly available due to privacy reasons.
